# Interplay between ER Ca^2+^ Binding Proteins, STIM1 and STIM2, Is Required for Store-Operated Ca^2+^ Entry

**DOI:** 10.3390/ijms19051522

**Published:** 2018-05-19

**Authors:** Heather A. Nelson, Colin A. Leech, Richard F. Kopp, Michael W. Roe

**Affiliations:** 1 Department of Cell and Developmental Biology, SUNY Upstate Medical University, Syracuse, NY 13210, USA; nelsonh@upstate.edu; 2 Department of Surgery, SUNY Upstate Medical University, Syracuse, NY 13210, USA; leechc@upstate.edu; 3 Department of Medicine, SUNY Upstate Medical University, Syracuse, NY 13210, USA; koppri@upstate.edu

**Keywords:** stromal interaction molecule, STIM1, STIM2, calcium (Ca^2+^), store-operated calcium entry, calcium signal transduction

## Abstract

Store-operated calcium entry (SOCE), a fundamentally important homeostatic and Ca^2+^ signaling pathway in many types of cells, is activated by the direct interaction of stromal interaction molecule 1 (STIM1), an endoplasmic reticulum (ER) Ca^2+^-binding protein, with Ca^2+^-selective Orai1 channels localized in the plasma membrane. While much is known about the regulation of SOCE by STIM1, the role of stromal interaction molecule 2 (STIM2) in SOCE remains incompletely understood. Here, using clustered regularly interspaced short palindromic repeats -CRISPR associated protein 9 (CRISPR-Cas9) genomic editing and molecular imaging, we investigated the function of STIM2 in NIH 3T3 fibroblast and αT3 cell SOCE. We found that deletion of *Stim2* expression reduced SOCE by more than 90% in NIH 3T3 cells. STIM1 expression levels were unaffected in the *Stim2* null cells. However, quantitative confocal fluorescence imaging demonstrated that in the absence of *Stim2* expression, STIM1 did not translocate or form punctae in plasma membrane-associated ER membrane (PAM) junctions following ER Ca^2+^ store depletion. Fluorescence resonance energy transfer (FRET) imaging of intact, living cells revealed that the formation of STIM1 and Orai1 complexes in PAM nanodomains was significantly reduced in the *Stim2* knockout cells. Our findings indicate that STIM2 plays an essential role in regulating SOCE in NIH 3T3 and αT3 cells and suggests that dynamic interplay between STIM1 and STIM2 induced by ER Ca^2+^ store discharge is necessary for STIM1 translocation, its interaction with Orai1, and activation of SOCE.

## 1. Introduction

Dynamic changes in the concentration of free calcium ions within the cytosol ([Ca^2+^]_c_) are universal intracellular signals that act over a wide temporal and spatial range to control many cell functions. Ca^2+^ signals are generated by the mobilization of calcium into the cytosol via influx through Ca^2+^-permeable ion channels in the plasma membrane (PM) and following release of Ca^2+^ from sites of intracellular sequestration.

Store-operated Ca^2+^ entry (SOCE), a specific type of Ca^2+^ influx mechanism, is an important Ca^2+^ signaling pathway in both excitable and non-excitable cells that is activated following release of Ca^2+^ sequestered within lumen of the endoplasmic reticulum (ER). SOCE provides local and global Ca^2+^ signals that regulate Ca^2+^-dependent biochemical events and is a source of Ca^2+^ for refilling depleted ER Ca^2+^ stores following cellular stimulation. The SOCE mechanism, also called capacitative Ca^2+^ entry, was first proposed by James Putney in 1986, but the molecular basis of SOCE was not discovered until 2005 and 2006 [1]. In siRNA screens, independent laboratories identified stromal interaction molecule 1 (STIM1) as an ER Ca^2+^ sensor responsible for the activation of SOCE [2,3]. Shortly after this discovery, genome-wide RNAi screens revealed that Orai1 was the ion-conducting pore subunit of store-operated channels (SOCs) [4,5].

Following the identification of STIM1 and Orai1 as the pivotal regulatory components of SOCE, numerous studies of a variety of cell types have led to the formulation of a consensus model that describes the mechanisms underlying the activation and inactivation of SOCE. In unstimulated, resting cells, STIM1 is diffusely distributed throughout the ER membrane, where it exists either as a monomer or as dimers [6,7,8,9]. Subsequent to exposure of cells to stimuli that cause release of ER Ca^2+^, the reduction in ER Ca^2+^ concentration ([Ca^2+^]_ER_) leads to the dissociation of Ca^2+^ from the Ca^2+^-binding helix-loop-helix structural domain (EF-hand motif) of STIM1, which consequently induces a conformational change and oligomerization of STIM1. This is followed by a rapid translocation and accumulation of STIM1 in discrete multi-protein clusters or punctae in plasma membrane-associated ER (PAM) nanodomains, subcellular regions in which the PM and ER membranes are in close apposition and functionally interconnected [3,10]. Current evidence suggests that within PAM sites, STIM1 binds to and activates Orai1 Ca^2+^ channels [3,6,11,12] via an interaction between a cytosolic motif of STIM1 known as the STIM1 Orai activating region (SOAR) and a carboxy terminus α-helical region of Orai1 [13,14,15].

STIM2, an ER single-pass transmembrane protein and homologue of STIM1, has also beenidentified, but relatively little is known about the roles of STIM2 in SOCE and other cellular functions. STIM1 and STIM2 are largely conserved and share high amino acid sequence homology. They both have a Ca^2+^-binding EF-hand domain, a sterile α motif (SAM) domain, three sequential coiled coil (CC) domains containing the SOAR, and a carboxy terminus lysine-rich domain [16]. It is of note, however, that the STIM proteins diverge considerably in the carboxy terminus half of the cytoplasmic domain, suggesting potential functional differences. Additionally, the Ca^2+^ binding affinity and activation kinetics of STIM2 differ from that of STIM1 [17,18,19]. STIM2 exhibits a lower Ca^2+^ affinity than STIM1. In a Ca^2+^-unbound state, the STIM2 EF-SAM domain is much more stable as a monomer; unlike STIM1, STIM2 does not readily aggregate or form puncta. Furthermore, a single amino acid difference in the STIM2 SOAR domain makes it a poor activator of Orai1 compared to STIM1 [20]. These differences in critical functional domains may contribute to alternate functions of the STIM proteins. The literature suggests that STIM2 functions as a homeostatic regulator of cytosolic and ER Ca^2+^ content, while it has only a minor contribution to SOCE in some cell types and none in others [2,3,17,21]. STIM2 has been shown to be active subsequent to small amplitude decreases in [Ca^2+^]_ER_ [17,21,22,23,24]. In sharp contrast, stable overexpression of STIM2 in HEK293 cells inhibits SOCE [25]. 

In order to provide a greater understanding of how STIM2 participates in Ca^2+^ homeostasis and signaling, we used clustered regularly interspaced short palindromic repeats (CRISPR)-Cas9 genomic editing and molecular imaging to determine the roles of STIM2 in Ca^2+^ homeostasis and SOCE in NIH 3T3 cells, a fibroblast cell line derived from mice, and αT3 cells, an immortalized gonadotroph cell line from mice. Our hypothesis was that STIM2 would be important for both Ca^2+^ homeostasis and SOCE. Our studies confirmed that STIM2 participates in the regulation of basal [Ca^2+^]_c_ in unstimulated NIH 3T3 and αT3 cells, and is necessary for activation of SOCE following depletion of ER Ca^2+^ stores. We also found that STIM2 was required for STIM1 translocation and puncta formation, as well as essential for the interaction of STIM1 with Orai1 in NIH 3T3 cells. Taken together, our findings suggest STIM2 recruits STIM1 and stabilizes it in PAM nanodomains to facilitate interaction with and activation of Orai1 channels.

## 2. Results

### 2.1. Store-Operated Ca^2+^ Entry in NIH 3T3 Cells

**Figure 1:**
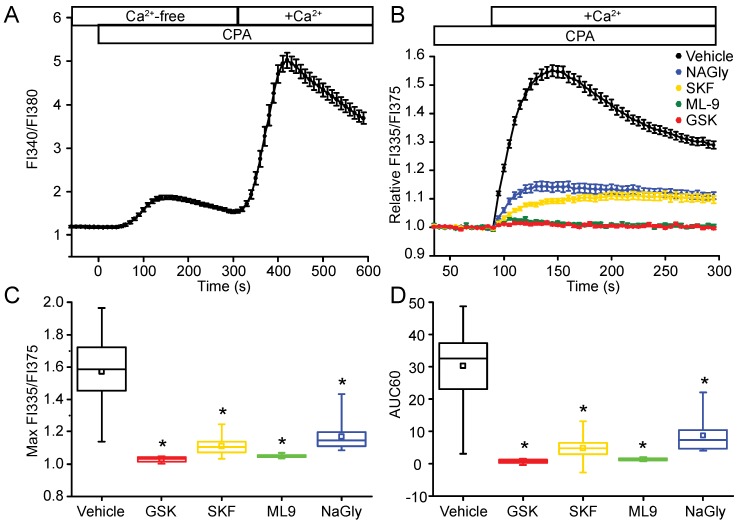
Store-operated Ca^2+^ entry in NIH 3T3 cells. (**A**) Store-operated Ca^2+^ entry (SOCE) in fibroblasts following passive endoplasmic reticulum (ER) Ca^2+^-store depletion with cyclopiazonic acid (CPA), an inhibitor of sarco/endoplasmic reticulum Ca^2+^-ATPase (SERCA). Cells grown on glass coverslips were loaded with Fura-2 and perifused with test solutions at 37 °C. Cells were initially bathed in standard extracellular solution (SES) containing 1 mM Ca^2+^ to establish a baseline. The bath solution was changed to a Ca^2+^-free SES (open bar; Ca^2+^-free), followed by Ca^2+^-free SES with 20 μM CPA to discharge and prevent refilling of the intracellular Ca^2+^ stores. SOCE was triggered by the subsequent application of SES with 1 mM Ca^2+^ (open bar; +Ca^2+^) in the continued presence of CPA; the rise of cytosolic [Ca^2+^] indicative of SOCE in single cells was measured using quantitative fluorescence microscopy and is expressed as the increase in the ratio of Fura-2 FI340/FI380. Data are expressed as the mean ± SEM (*n* = 67 cells). (**B**) Pharmacological analysis of SOCE. Cells were loaded with Fura-2 in SES and Ca^2+^ was measured with the FlexStation 96-well plate reader. The cells were incubated in a Ca^2+^-free SES containing CPA (20 μM) and the SOCE inhibitor or vehicle control. Application of GSK-7975A (GSK; 50 μM), SKF96365 hydrochloride (SKF; 10 μM), ML-9 (100 μM), or NAGly (30 μM) before the reintroduction of extracellular Ca^2+^ (open bar; +Ca^2+^) caused a significant reduction in the (**C**) normalized peak amplitude (Max FI335/FI375) and (**D**) area under the curve (AUC60) of the store-operated Ca^2+^ response. Graph data in (**B**) are plotted as the time-dependent change in the mean ± SEM of the fold change in the ratio of Relative FI335/FI375, averaged from 12 or more wells for each inhibitor from at least three independent experiments. In the box and whisker plots, the center solid line marks the median, small open square within the box depicts the mean, the ends of the box are the 25th and 75th quartiles, and whiskers are the minimum and maximum measured values. * *p* < 0.05 compared to vehicle control.

Depletion of the intracellular Ca^2+^ stores with cyclopiazonic acid (CPA), an inhibitor of sarcoendoplasmic reticulum Ca^2+^-ATPase (SERCA), followed by addition of extracellular Ca^2+^, resulted in an increase in [Ca^2+^]_c_, characteristic of SOCE found in many cell types (Figure 1A). Pharmacological inhibitors were used to characterize some of the mechanisms underlying SOCE in the NIH 3T3 cells. Application of [2,6-difluoro-*N*-(1-[4-hydroxy-2-(trifluoromethyl)benzyl]-1*H*-pyrazol-3-yl)benzamide] (GSK-7975A), a reagent that inhibits SOCE downstream of STIM1 oligomerization and STIM1-Orai1 interaction [26], attenuated the maximum amplitude of the rise in [Ca^2+^]_c_ during the first 60 s of Ca^2+^ addback by 95 ± 3% (Figure 1B,C). The overall time-dependent SOCE response, measured by curve integration analysis of the initial 60-s period (area under the Ca^2+^ response curve, AUC60) during the incubation of cells in Ca^2+^-containing solutions was reduced by 97 ± 2% (Figure 1B,D). SKF96365 hydrochloride, a non-specific SOCE inhibitor that inhibits Ca^2+^ influx [3,27], reduced maximum amplitude of SOCE by 81 ± 8% and reduced the AUC60 by 84 ± 9% (Figure 1B,D). A structurally unrelated reagent, 1-(5-chloronaphthalene-1-sulfonyl)-1H-hexohydro-1,4-diazepine (ML-9), an inhibitor of myosin light chain kinase that interferes with STIM1 translocation and puncta formation [28], also attenuated SOCE. The maximum [Ca^2+^]_c_ rise and overall SOCE response was reduced by 91 ± 2% and 95 ± 1%, respectively (Figure 1B,D). *N*-arachidonoyl glycine (NAGly), an endocannabinoid that reversibly inhibits SOCE by disrupting STIM1-Orai1 interactions [29], also partially inhibited SOCE in NIH 3T3 cells; the peak increase in cytosolic [Ca^2+^] and AUC60 were reduced by 70 ± 2% and 71 ± 2%, respectively (Figure 1B,D). 

**Figure 2:**
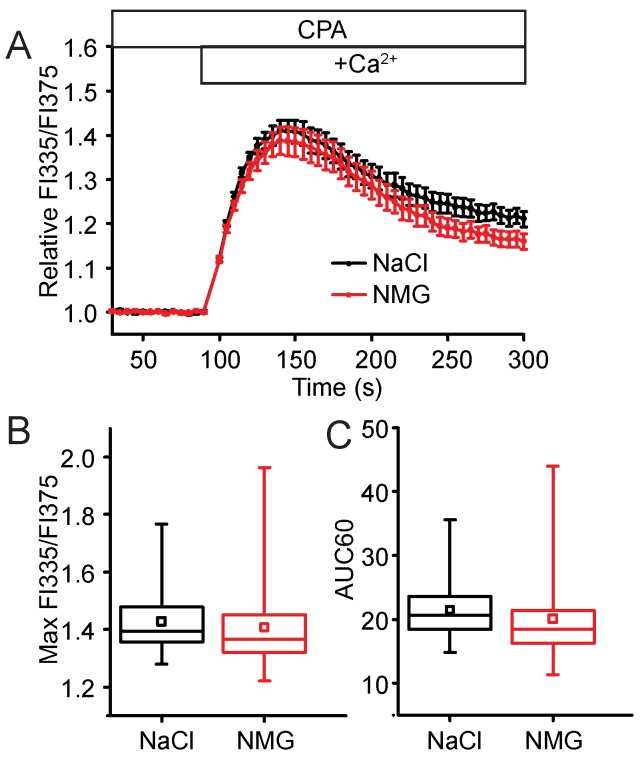
SOCE occurs through a highly Ca^2+^-selective channel. (**A**) Block of non-selective cation channels by ionic substitution in SES does not affect SOCE in NIH 3T3 cells. Cells in a 96-well plate were loaded with Fura-2 and then washed with Ca^2+^-free SES containing 20 μM CPA (open bar) and 140 mM NaCl or 140 mM *N*-methyl-d-glucamine hydrochloride (NMG). The plate was placed in a FlexStation 3, and readings were taken every 5 s. After 95 s, Ca^2+^-containing SES (open bar; +Ca^2+^) with NaCl or NMG was injected into each well. Data show the mean ± SEM of the Relative FI335/FI375, averaged across 24 wells for each solution from three independent experiments. (**B**) The normalized maximum SOCE amplitude (Max FI335/FI375) and (**C**) AUC of the Ca^2+^ influx during the first 60 s after Ca^2+^ add back (AUC60).

**Figure 3:**
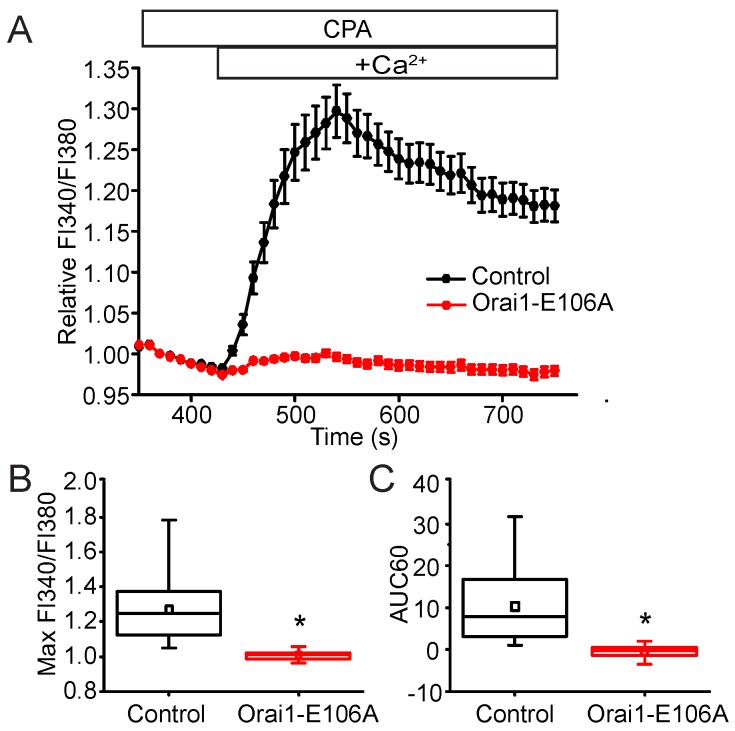
Orai1 carries the store-operated current in NIH 3T3 cells. (**A**) Suppression of Orai1 activity ablates SOCE in fibroblasts. Cells were transfected with an empty DsRed vector (control) or a dominant-negative Orai1 mutant (Orai-E106A) and DsRed. Two days post-transfection, cells were loaded with Fura-2 and perifused with test solutions at 37 °C. Following depletion of the ER Ca^2+^ stores with 20 μM CPA (open bar) in Ca^2+^-free extracellular solutions, bathing cells in a solution with 1 mM Ca^2+^ (open bar; +Ca^2+^) initiated a SOCE-mediated rise in [Ca^2+^]_c_ that was almost completely absent in cells expressing Orai1-E106A. Data are expressed as mean ± SEM Relative FI340/FI380 from three or more independent experiments (*n* = 30 control cells, *n* = 68 Orai1-E106A cells). (**B**) The normalized maximum FI340/FI380 amplitude and (**C**) AUC60 of the Ca^2+^ influx from individual cells. * *p* < 0.05 compared to control.

In most types of cells, Orai1, a highly selective Ca^2+^ channel, is considered to be the primary SOC channel activated by STIM1 in response to ER Ca^2+^ store depletion [30]. In addition to the Orai proteins, members of the transient receptor potential canonical (TRPC) ion channel family have also been shown to be involved in Ca^2+^ entry in response to Ca^2+^-store depletion [31,32]. Unlike Orai channels, TRPC channels are Ca^2+^-permeable non-selective cation channels [33,34]. Ionic substitution in extracellular bathing solutions was used to characterize the Ca^2+^-selectivity of the channels mediating SOCE in the NIH 3T3 cells. Replacement of external Na^+^ with an equimolar concentration of *N*-methyl-d-glucamine (NMG), a cation that enters and blocks the ion-conducting pore of non-selective channels, but not Ca^2+^ selective channels, had no effect on SOCE. This suggests that SOCE is mediated by a channel that exhibits Ca^2+^ selectivity in the NIH 3T3 fibroblasts (Figure 2). Our findings suggest that Orai1 is responsible for the SOCE in NIH 3T3 fibroblasts. To further test the hypothesis that Orai1 specifically is responsible for the SOCE response we were measuring, a dominant-negative Orai1 mutant, Orai1 E106A, was transiently expressed in the cells. In cells expressing Orai1 E106A, SOCE was nearly completely abolished, consistent with Orai1 playing a predominant role in SOCE (Figure 3). Since we were able to establish that SOCE occurs through Orai1 and can be attenuated by several SOCE inhibitors, we next sought to identify the role STIM2 plays in this process.

### 2.2. STIM2 Is Expressed and Regulates Intracellular [Ca^2+^]_c_ in NIH 3T3 Cells

**Figure 4:**
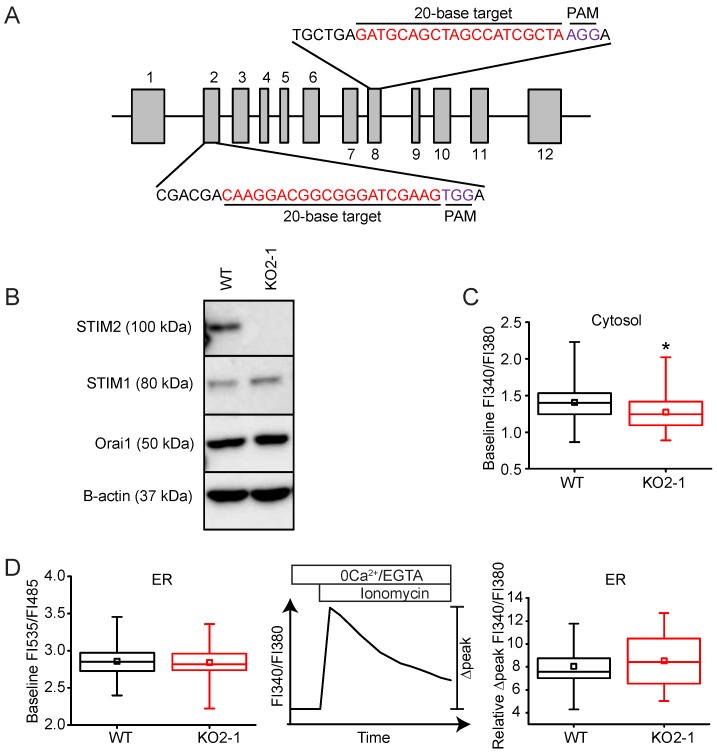
STIM2 knock-out in NIH 3T3 cells does not alter Ca^2+^ homeostasis. (**A**) Exon map of murine *Stim2* showing the 20-base-pair gRNA CRISPR-Cas9 targets in exons 2 and 8 used in the studies. (**B**) Western immunoblot of whole cell protein lysates (20 μg) harvested from control (WT) or *Stim2*-null (KO2-1) NIH 3T3 cells. Lysates were resolved by 8% SDS-PAGE and identified using selective antibodies against STIM2, STIM1, Orai1, and β-actin. (**C**) Baseline FI340/FI380 in WT (*n* = 172 cells) and KO2-1 (*n* = 294 cells) NIH 3T3 fibroblasts. * *p* < 0.05 compared to WT (**D**) STIM2 KO did not alter basal [Ca^2+^]_ER_ as measured by two independent methods. Left Panel: Comparison of [Ca^2+^]_ER_ in WT (*n* = 125 cells) and KO2-1 (*n* = 101 cells) cells using D1ER, a genetically-encoded biosensor of ER Ca^2+^. Middle Panel: Response of [Ca^2+^]_c_ following release of intracellular Ca^2+^ stores with ionomycin (2 µM). Right Panel: The increase in cytosolic Ca^2+^ (Relative ∆peak FI340/FI380) following ionomycin-induced discharge of ER Ca^2+^ in the *Stim2* null cells was similar to that measured in control cells (KO2-1: *n* = 62 cells; WT: *n* = 61 cells).

STIM2 has been shown to be a regulator of Ca^2+^ homeostasis in HeLa, HUVEC, and HEK293T cells [17]. Whether STIM2 plays a similar role in other types of cells remains unclear. The involvement of *STIM2* in Ca^2+^ homeostasis and signaling was investigated by knocking out its expression in NIH 3T3 fibroblasts using CRISPR-Cas9-mediated genomic editing. STIM2 is expressed in NIH 3T3 cells, and its expression was completely eliminated (KO2-1) by targeting a sequence in exon 2 (Figure 4A,B). Cells undergoing the same transfection process, but showing no loss of STIM2 expression, were used as controls (WT). The expression of STIM1 was not affected by STIM2 knock-out; however, Orai1 expression was modestly elevated in STIM2 KO2-1 cells (Figure 4B). Since STIM2 has been reported to be a regulator of basal [Ca^2+^], we investigated whether STIM2 knock-out would alter cytosolic Ca^2+^ homeostasis in unstimulated cells. We found that loss of STIM2 reduced resting [Ca^2+^]_c_ in STIM2 KO2-1 NIH 3T3 cells (FI340/FI380: 1.27 ± 0.23, *n* = 294) compared to WT cells (FI340/FI380: 1.40 ± 0.22, *n* = 172, *p* < 0.05) (Figure 4C). 

We also measured [Ca^2+^]_ER_ using D1ER, a fluorescence resonance energy transfer (FRET)-based indicator of [Ca^2+^]_ER_ [35]. Unlike previous reports, we saw no difference (*p* > 0.05) between the resting level of [Ca^2+^]_ER_ in STIM2 knockout (FRET FI535/FI485: 2.8 ± 0.2, *n* = 101) and control (FRET FI535/FI485: 2.9 ± 0.2, *n* = 125) cells (Figure 4D). As an alternative method for measuring the basal level of Ca^2+^ in the ER stores, we used Fura-2 to measure the change in the cytosolic [Ca^2+^] peak following addition of ionomycin, a membrane permeable Ca^2+^ ionophore. Cells were bathed in a Ca^2+^-free extracellular solution supplemented with ethylene glycol-*bis*(β-aminoethyl ether)-*N*,*N*,*N*′,*N*′-tetraacetic acid (EGTA) to prevent the influx of extracellular Ca^2+^ across the plasma membrane. The ER is the major Ca^2+^ store in the cell; therefore, the normalized maximum Fura-2 FI340/FI380 (∆peak) of the Ca^2+^ response following application of ionomycin is an indirect measure of the amount of Ca^2+^ sequestered within the lumen of the ER. Using this approach, we found no difference in ER Ca^2+^ store content in STIM2 null cells (∆peak: 8.5 ± 2.2, *n* = 68) compared to WT (Δpeak: 8.1 ± 1.6, *n* = 61) (Figure 4D). Taken together, our data suggest that knockout of STIM2 modestly reduces basal cytosolic [Ca^2+^], but not ER Ca^2+^ store content in NIH 3T3 cells. 

### 2.3. STIM2 Is an Important Regulator of SOCE

**Figure 5:**
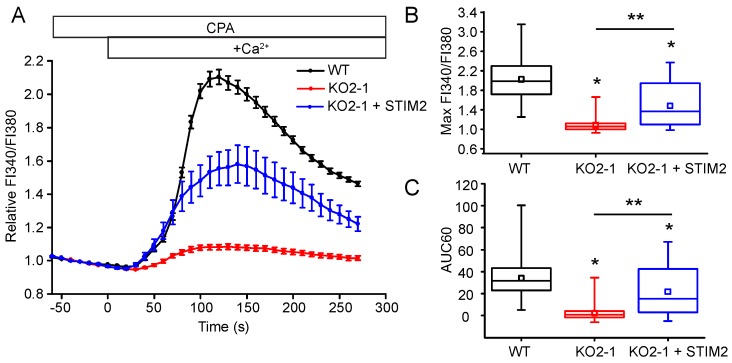
STIM2 regulates SOCE in NIH 3T3 fibroblasts. (**A**) Single-cell analysis of SOCE in WT and KO2-1 NIH 3T3 cells. STIM2 expression was reconstituted by transient transfection of YFP-STIM2 in the STIM2 null cells (KO2-1 + hSTIM2). Note that SOCE was partially restored by STIM2 reconstitution. The data are expressed as the mean ± SEM FI340/FI380 (*n* = 75 WT cells, *n* = 74 KO2-1 cells, *n* = 22 KO2-1 + STIM2 cells). (**B**) The normalized maximum FI340/FI380 amplitude, and (**C**) AUC60 of Ca^2+^ influx from individual cells. * *p* < 0.05 compared to WT, ** *p* < 0.05 compared to KO2-1.

Whether STIM2 regulates SOCE is not well established. Some studies have indicated that STIM2 does not play a role in SOCE, while others have found it has a minor contribution to SOCE [2,21,22,24,36,37,38,39,40]. In contrast, STIM2 has been found to be the primary regulator of SOCE in mouse cortical neurons and *Xenopus* oocytes [41,42]. However, in rat cortical neurons, STIM1 was critical for SOCE, while STIM2 was important for regulation of resting [Ca^2+^]_ER_ [43]. In comparison with control cells, the maximum [Ca^2+^]_c_ amplitude and overall SOCE response was significantly reduced (*p* < 0.05) in the NIH 3T3 STIM2 knockout cells (KO2-1) (maximum FI340/FI380: 1.1 ± 0.1, AUC60: 2.2 ± 7.1, *n* = 74) compared to WT (maximum FI340/FI380: 2.0 ± 0.4, AUC60: 34.0 ± 15.3, *n* = 75) (Figure 5). The inhibition of SOCE in the NIH 3T3 STIM2 KO2-1 cells was partially restored following reconstitution of STIM2 protein levels with human STIM2 (maximum FI340/FI380: 1.5 ± 0.4, AUC60 21.6 ± 22.8, *n* = 22) (Figure 5). 

**Figure 6:**
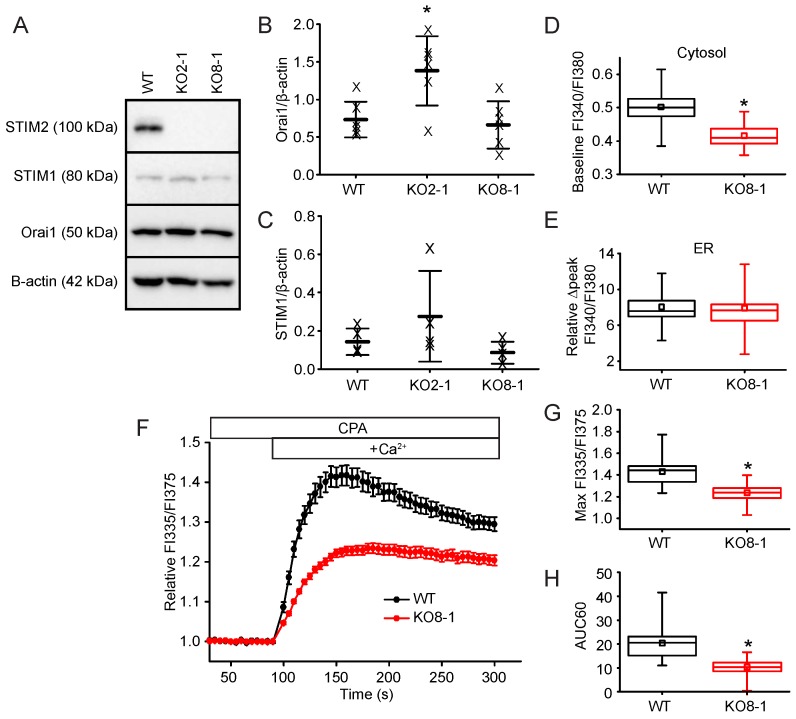
Characterization of *Stim2* knock-out in NIH 3T3 KO8-1 cells. (**A**) Western immunoblot of whole cell protein lysates (20 μg) harvested from control (WT) or *Stim2*-null (KO2-1 and KO8-1) NIH 3T3 cells. Lysates were resolved by 8% SDS-PAGE and identified using selective antibodies against STIM2 and β-actin. (**B**) Although Orai1 expression was elevated in KO2-1 (* *p* < 0.05 compared to WT), Orai1 expression was not altered in KO8-1 as determined by quantification of Orai1 band intensity normalized to the band intensity of β-actin from multiple western immunoblots. Each “X” represents the ratio from one independent blot and protein lysate. Data are represented as mean (center thick line) ± SD (error bars). (**C**) STIM1 expression is unchanged in both clones of *Stim2* null cells. (**D**) Basal [Ca^2+^]_c_ (Baseline FI340/FI380) in WT (*n* = 90 cells) and KO8-1 (*n* = 85 cells) NIH 3T3 fibroblasts. (**E**) STIM2 KO did not alter basal [Ca^2+^]_ER_ in STIM2 KO8-1 (*n* = 62 cells) compared to WT (*n* = 61 cells) as measured by the increase in cytosolic Ca^2+^ (Relative ∆peak FI340/FI380) following ionomycin-induced discharge of ER Ca^2+^. (**F**) FlexStation analysis of SOCE in WT and KO8-1 NIH 3T3 cells. The data are expressed as the mean ± SEM Relative FI335/FI375 (*n* = 30 WT wells, *n* = 62 KO8-1 wells). (**G**) The normalized maximum FI335/FI375 amplitude and (**H**) AUC60 of the SOCE-mediated Ca^2+^ influx from individual wells. * *p* < 0.05 compared to WT.

To confirm that the phenotype we were studying in the STIM2 KO2-1 clone was a result of STIM2 knockout and not due to CRISPR-Cas9 off-target effects or changes related to clonal selection, we knocked out STIM2 in another NIH 3T3 cell clone by targeting a different exon, exon 8, in *Stim2* (Figure 4A) to generate KO8-1 (Figure 6A). Orai1 and STIM1 protein expression levels were unchanged in the NIH 3T3 KO-8-1 *Stim2* null cells (Figure 6A–C). In agreement with our findings from KO2-1, resting [Ca^2+^]_c_ in KO8-1 cells was 16% lower than in control cells (KO8-1: FI340/FI380: 0.42 ± 0.03, *n* = 85 cells; WT: FI340/FI380: 0.50 ± 0.04, *n* = 90 cells; *p* < 0.05) (Figure 6D). Similarly, we saw no change in ER Ca^2+^ content as measured by the ∆peak of the Ca^2+^ response to ionomycin in 0 Ca^2+^/EGTA (KO8-1 ∆peak: 7.9 ± 1.8, *n* = 62; WT ∆peak: 8.1 ± 1.6, *n* = 61, *p* > 0.05) (Figure 6E). To complement the reconstitution experiments in confirming a specific role for STIM2 in SOCE in KO2-1, we also measured the store-operated response in our KO8-1 clone. In KO8-1, the normalized maximum [Ca^2+^]_c_ amplitude of the SOCE response was significantly reduced by 45% (maximum FI335/FI375: 1.2 ± 0.2, *n* = 62 wells) compared to WT (maximum FI335/FI375: 1.4 ± 0.4, *n* = 30 wells) (Figure 6F,G). The overall SOCE response measured by AUC60 was reduced by 50% (WT: AUC60: 20.4 ± 6.8, *n* = 30 wells; KO8-1: AUC60: 10.1 ± 3.3, *n* = 62 wells) (Figure 6H). These data confirm the validity of our NIH 3T3 STIM2 KO2-1 clone and confirm that loss of STIM2 results in a modest reduction in basal [Ca^2+^]_c_, no change in [Ca^2+^]_ER_, and reduced SOCE. 

**Figure 7:**
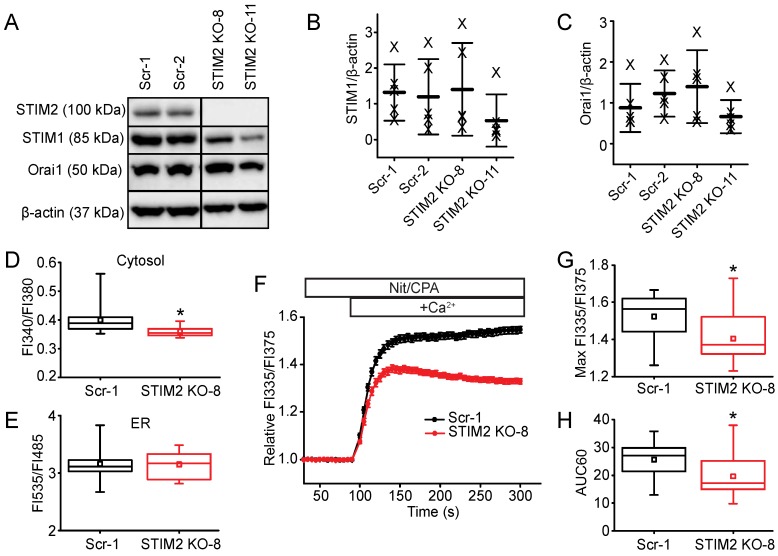
Knock-out and characterization of STIM2 function in αT3 cells. (**A**) Western immunoblot of whole cell protein lysates (20 μg) harvested from two separate clones of control (Scr-1 and Scr-2) or *Stim2* knock-out (KO-8 and KO-11) αT3 cells. Lysates were resolved by 8% SDS-PAGE and identified using selective antibodies against STIM2, STIM1, Orai1, and β-actin. The vertical line represents separation of the lysates on the same blot that was cut out for the figure presentation. The same blot and exposure time were used for both sides of the image. (**B**) Quantification of STIM1 band intensity normalized to the band intensity of β-actin, showing no significant difference in STIM1 protein expression in *Stim2* null clones (STIM2 KO-8 and KO-11) compared to controls (Scr-1 and Scr-2). Each “X” represents the ratio from one independent blot. Data are represented as mean (center thick line) ± SD (error bars). (**C**) Quantification of Orai1 band intensity normalized to that of β-actin, showing no change in Orai1 protein expression in *Stim2* null clones (STIM2 KO-8 and KO-11) compared to controls (Scr-1 and Scr-2. (**D**) Basal [Ca^2+^]_c_ in unstimulated resting Scr-1 (*n* = 59) and KO-8 (*n* = 20) αT3 cells (FI340/FI380). (**E**) Comparison of basal [Ca^2+^]_ER_ in Scr-1 (*n* = 34) and KO-8 (*n* = 18) αT3 cells (FI535/FI485). (**F**) FlexStation 3 analysis of the SOCE in αT3 cells comparing Scr-1 (*n* = 48 wells) to *Stim2* KO-8 (*n* = 54 wells). Data show the mean ± SEM of the Relative FI335/FI375. (**G**) The normalized maximum FI335/FI375 amplitude, and (**H**) AUC60 of the SOCE response. * *p* < 0.05 compared to WT.

To determine the role of STIM2 in a different cell type, we deleted the expression of STIM2 in αT3 cells, a mouse pituitary cell line, using CRISPR-Cas9 gene editing technology (Figure 7A). There was no change in the expression of STIM1 or Orai1 in the STIM2 null neuroendocrine cells (Figure 7A–C). Resting, basal [Ca^2+^]_c_ in the *Stim2* null αT3 cells was 10% lower (FI335/FI375: 0.36 ± 0.17, *n* = 20 wells) than in control cells expressing a scrambled gRNA sequence (Scr-1) (FI335/FI375: 0.40 ± 0.04, *n* = 59 wells), and there was no difference in basal [Ca^2+^]_ER_ (Figure 7D,E). Additionally, we found a significant reduction in the maximum [Ca^2+^]_c_ amplitude and AUC60 of the SOCE response in STIM2 null cells (normalized maximum FI335/FI375: 1.4 ± 0.1; AUC60: 19.5 ± 6.2, *n* = 54 wells) compared to control cells (normalized maximum FI335/FI375: 1.5 ± 0.1; AUC60: 26.7 ± 6.1, *n* = 48 wells) (Figure 7F–H). Multiple clones of STIM2 KO and control (expressing scrambled gRNA sequences) αT3 cells were evaluated: all exhibited phenotypes similar to STIM2 KO-8 and Scr-1, respectively. Taken together, our data suggests an important role for STIM2 in regulating cytosolic Ca^2+^ homeostasis and SOCE in both excitable and non-excitable cell lines. 

### 2.4. STIM1 Translocation

**Figure 8:**
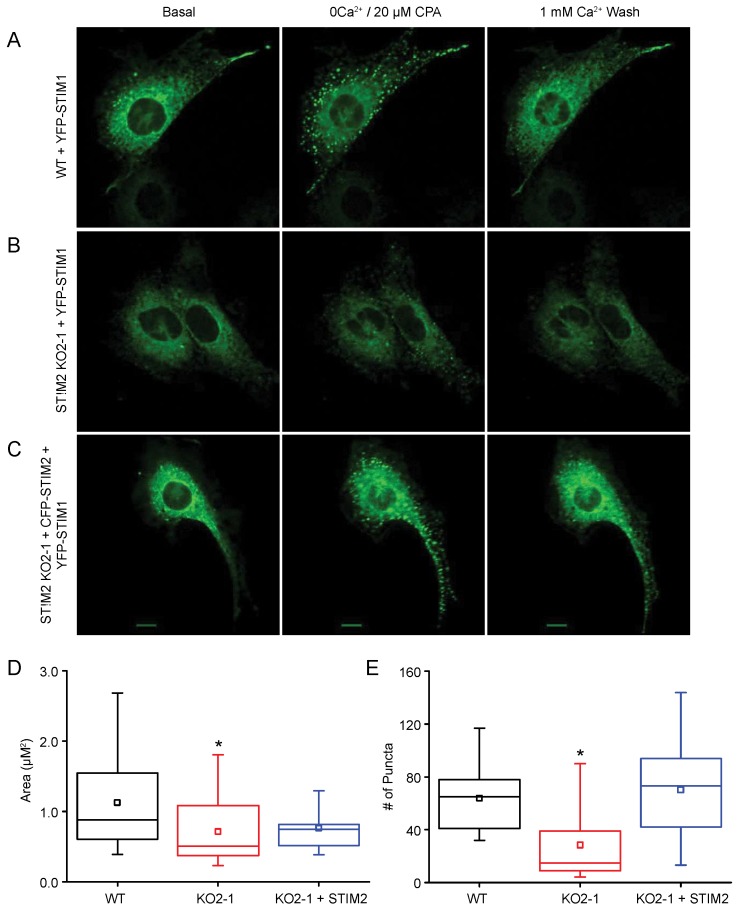
STIM2 is required for STIM1 puncta formation. Subcellular localization of YFP-STIM1 in NIH 3T3 (**A**) WT, (**B**) KO2-1, or (**C**) KO2-1 cells transiently expressing CFP-STIM2. Cells were transfected with YFP-STIM1. Representative confocal images of cells at rest (left panels; Basal), after depleting ER stores with CPA in a Ca^2+^-free solution (center panels; 0Ca^2+^/20 μM CPA), and after re-administration of extracellular Ca^2+^ (right panels; 1 mM Ca^2+^ Wash). Scale bar = 10 μm. (**D**) Average area of individual puncta, and (**E**) average number of puncta per cell in ER Ca^2+^ store-depleted cells. Data were recorded from at least six independent experiments for each group of cells: WT (*n* = 19 cells), KO2-1 (*n* = 18 cells), and STIM2 reconstituted KO2-1 (*n* = 9 cells). * *p* < 0.05 compared to WT.

We used high-resolution fluorescence confocal imaging with STIM1 fused to yellow fluorescent protein (YFP-STIM1) to characterize the distribution of STIM1 before and after store depletion. Confirming previous reports [6,9,44], YFP-STIM1 translocated and formed puncta near the PM following CPA–induced store depletion and returned to a diffuse distribution after the CPA was removed and the ER Ca^2+^ stores refilled (Figure 8A). In contrast, in the NIH 3T3 STIM2 KO2-1 cells, YFP-STIM1 did not form puncta to the same extent as in control cells (Figure 8B). The puncta in *Stim2* null cells were significantly smaller. Quantification of puncta number and area revealed that puncta number per cell was reduced by 46% in the *Stim2* null cells, and the average area of each punctum was significantly reduced by 37% (WT: 1.13 ± 0.68 μm^2^, *n* = 19; KO2-1: 0.71 ± 0.47 μm^2^, *n* = 18) (Figure 8D,E). When STIM2 expression was reconstituted by transient overexpression of human STIM2 (hSTIM2), puncta number was fully reconstituted and puncta area was increased by 6% (KO2-1 + hSTIM2: 0.76 ± 0.33 μm^2^, *n* = 9) (Figure 8C–E). This suggests that STIM2 is required for efficient translocation and clustering of STIM1 molecules in PAM nanodomains of NIH 3T3 cells. 

### 2.5. STIM2 Facilitates STIM1 Interaction with Orai1

**Figure 9:**
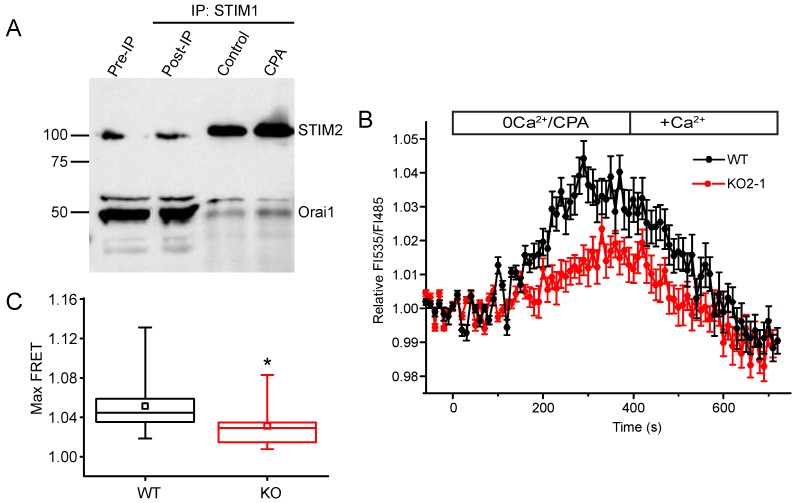
STIM2 facilitates STIM1-Orai1 interaction. (**A**) Immunoprecipitation studies using NIH 3T3 cell extracts under store-filled (Control) or store-depleted (CPA) conditions. Immunoprecipitation was performed using anti-STIM1 antibody followed by immunoblotting with anti-STIM2 and anti-Orai1 antibodies. The STIM1 antibody pulled down STIM2 and Orai1 under both conditions; however, the interaction was enhanced in store-depleted conditions. The input protein (Pre-IP) and the protein remaining after pull-down (Post-IP) are shown. Molecular mass markers are indicated. (**B**) Changes in FRET between YFP-STIM1 and CFP-Orai1 as intracellular Ca^2+^ stores were depleted (open bar; 0Ca^2+^/CPA) and then refilled (open bar; +Ca^2+^) in WT (black line) and STIM2 KO2-1 (red line). The data are expressed as the mean ± SEM fold-change in FRET FI535/FI485 relative to FI535/FI485 in resting unstimulated cells from five independent experiments (WT: *n* = 28 cells; KO2-1: *n* = 26 cells). FI535/FI485 in resting unstimulated cells was not different in KO2-1 compared to control. (**C**) The maximum values of fold-change in FRET FI535/FI485 (Max FRET). * *p* < 0.05 compared to WT.

One mechanism by which STIM2 could facilitate STIM1 clustering is through a direct interaction. STIM1–STIM2 interactions have been demonstrated in HeLa cells, human platelets, HEK293 cells, and mouse neurons [17,45,46]. To confirm an interaction of STIM1 with STIM2, we evaluated whether endogenous STIM1 and STIM2 interacts in NIH 3T3 cells using a co-immunoprecipitation assay. Whole cell lysates from control cells with fully filled ER Ca^2+^ stores under basal, unstimulated conditions and cells treated with CPA to deplete [Ca^2+^]_ER_ and activate SOCE were examined. A STIM1-specific antibody was used to pull down STIM1 and immunoprecipitates were analyzed by Western blot for STIM2 and Orai1. STIM2 was detected in the STIM1 immunoprecipitates in both the control and CPA-treated cell lysates; under ER store-depletion conditions, the interaction between STIM1 and STIM2 was enhanced (Figure 9A). Additionally, the level of Orai1 in the STIM1 immunoprecipitates was increased after depleting ER Ca^2+^ stores (Figure 9A). These findings strongly suggest that STIM1 and Orai1 interact in NIH 3T3 cells similar to that reported in other types of cells [45,47,48,49]. Since our studies suggested that STIM2 was required for STIM1 puncta formation in the NIH 3T3 cells, we hypothesized that the interaction between STIM1 and Orai1 would be reduced in STIM2 KO cells. To test this hypothesis, we used FRET imaging and found that FRET between YFP-STIM1 and cyan fluorescent protein (CFP)-Orai1 was reduced by 39% in the STIM2 null cells (Figure 9B,C). Taken together, our data suggests that STIM2 is required for STIM1 puncta formation and efficient clustering at ER-PM junctions where it interacts with and activates Orai1. 

## 3. Discussion

The role of STIM1 and Orai1 in SOCE has been well-defined and characterized [2,3,4,5,10,50]. Following ER store depletion, ER transmembrane Ca^2+^-sensor STIM1 oligomerizes and translocates to ER-PM junctions, where it forms puncta, interacts with and activates the pore forming channel, Orai1, to stimulate Ca^2+^influx [3,4,10,13,44,51,52]. The role for STIM2 in SOCE, however, is much more ambiguous. Silencing of STIM2 in HEK293, neutrophil-like HL-60 cells, rat pulmonary arterial smooth muscle, airway myocytes, and CD4 T^+^ cells did not affect SOCE [2,21,23,37,38,39]. In contrast, knockdown of STIM2 in HeLa cells, B cells, MDA-MB-231 cells, and mouse embryonic fibroblasts (MEFs) resulted in a small reduction of SOCE, while a more significant reduction in SOCE was seen when STIM2 was silenced in murine T cells, cortical neurons, mast cells, and *Xenopus* oocytes [3,21,22,40,41,42,53,54]. Even more surprising was the finding that STIM2 inhibited SOCE when stably overexpressed in HEK293, PC12, A7r5 and Jurkat T cells [23,25]. Here, we report a major role for STIM2 in regulating SOCE in NIH 3T3 cells and αT3 cells. Further analysis of store-depletion induced clustering of STIM1 and Orai1 showed that STIM2 is required for the recruitment of STIM1 to ER-PM junctions where STIM1 interacts with Orai1. Our data suggest that STIM2 facilitates STIM1 oligomerization and translocation to ER-PM junctions, and subsequently, activates SOCE following ER Ca^2+^ store depletion.

STIM2 has been demonstrated to play a role in maintaining basal Ca^2+^ homeostasis in mouse neurons, HeLa cells, HUVEC cells and HEK293T cells [17,41]. Our findings confirm this role of STIM2 in NIH 3T3 fibroblasts and αT3 cells, although the levels of cytosolic Ca^2+^ reduction in the STIM2 knockout cells were modestly reduced by 10–15% relative to control cells. The effects of silencing *Stim2* expression on Ca^2+^ homeostasis in other organelles is unclear. Previously, siRNA knockdown of *Stim2* revealed that basal [Ca^2+^]_ER_ in HeLa cells was reduced [17]. In contrast, knockdown of *Stim2* in HEK293 cells with siRNA had no effect on thapsigargin-induced release of Ca^2+^ [23]. Completely eliminating *Stim2* expression in NIH 3T3 and αT3 cells using CRISPR-Cas9 genomic editing has no impact on resting ER Ca^2+^ store content. The absence of any effect on ER Ca^2+^ levels in resting cells could reflect either differences in the role of *Stim2* in the regulation of ER Ca^2+^ homeostasis in different types of cells or an effect of partial knockdown of *Stim2* compared with a total knockout of the gene’s expression using CRISPR-Cas9. Additional work in other types of cells, both in vitro and in vivo are needed to more fully understand how STIM2 regulates Ca^2+^ homeostasis in subcellular compartments.

Because STIM2 is a weaker activator of Orai1, we wondered how loss of STIM2 might have such a large impact on SOCE. It was previously shown that at low-stimulus intensities, STIM2 enhances activation of SOCE by promoting STIM1 clustering [46]. Additionally, in rat hippocampal neurons, STIM1 did not co-localize with Orai1 in the absence of STIM2 [55]. Given these data, we hypothesized that in *Stim2* null cells, SOCE was reduced due to an inability of STIM1 to interact fully with Orai1. Indeed, we show YFP-STIM1 does not efficiently translocate and form puncta when overexpressed in STIM2 KO cells as quantified by reduced puncta number and area, suggesting a role for STIM2 in STIM1 puncta formation. Interestingly, we found this to be true even following maximal store-depletion induced by SERCA inhibition with CPA, not just at low stimulus intensities. Furthermore, our FRET imaging studies in living cells showed that STIM1 interaction with Orai1 was impaired in *Stim2* null cells. Taken together, these data suggest a critical role for STIM2 in facilitating the association of functional STIM1-Orai1 complexes.

**Figure 10:**
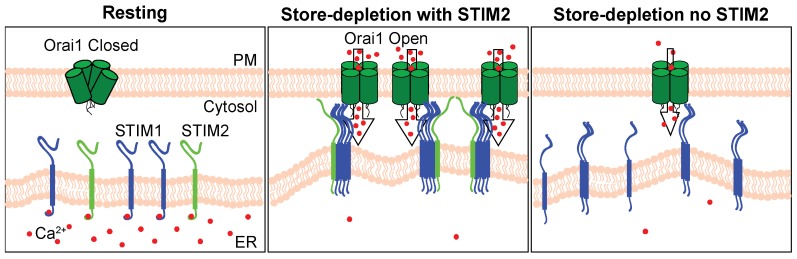
Model illustrating the importance of STIM2 in the clustering and recruitment of STIM1 to ER-PM junctions where it interacts with Orai1. At rest when the intracellular Ca^2+^ stores are full, the EF-hand domain of STIM1 and STIM2 bind Ca^2+^ and maintain an inactive confirmation (**left**). In WT cells containing STIM1 and STIM2, Ca^2+^ store-depletion causes Ca^2+^ dissociation from the EF-hand, initiating a destabilization-coupled oligomerization event. STIM2 provides a building block for large multimers of STIM1 to form. Given the STIM2 lysine-rich domain has a higher affinity for PM phospholipids, it likely plays a role in recruiting and stabilizing STIM1 at ER-PM junctions, where it interacts with and activates Orai1 (**center**). In the absence of STIM2, STIM1 cannot form large oligomers and is inefficient at interacting with and gating Orai1 (**right**).

Differences between our work and previously published data regarding a threshold of activation for STIM2 recruitment of STIM1 to ER-PM junctions is likely explained by differences in experimental approach used to silence *Stim2* expression. Many of the early studies used siRNA knockdown of STIM2 [2,21,23,37,42], yielding varying levels of efficacy, which could significantly impact the interpretation of the data. It is likely that partial knockdown of STIM2 expression may not be sufficient to reveal a role of STIM2 in SOCE. Our work, using CRISPR-Cas9 technology to completely ablate *Stim2* expression, indicated that STIM2 is required for STIM1 translocation, the interaction of STIM1 with Orai1, and activation of SOCE. We propose a model in which STIM2 is required for STIM1 puncta formation and activation of Orai1 (Figure 10). We suggest that when the ER Ca^2+^ stores are full, STIM1 and STIM2 are located diffusely within the ER. Following stimulation and store-depletion, STIM1 oligomerizes and associates with STIM2 through the conserved SAM and CC domains. The STIM2 EF-SAM domain is more stable than that of STIM1 [19], which may be important for its ability to stabilize large STIM1 oligomers. Furthermore, the lysine-rich (K-rich) domain present in the C-terminus of both STIM proteins binds to PM phosphatidylinositol 4,5-bisphosphate (PIP_2_) to stabilize STIM at ER-PM junctions [6,56,57]. STIM2 was shown to have a higher affinity for PM lipids than STIM1, suggesting that it may be a stronger stabilizer of PAM functional integrity and critical for supporting STIM1-dependent Ca^2+^ signaling in these junctions [57]. This may help STIM2 serve as an anchoring protein. Through strong electrostatic interactions with the PM, STIM2 is able to anchor and stabilize large STIM1 clusters in close proximity to Orai1. Additional studies will be necessary to better understand why in the absence of STIM2, STIM1 is not able to form large clusters and is inefficient at gating Orai1, resulting in the reduced SOCE. 

In conclusion, our data demonstrate that STIM2 is a critical regulator of SOCE in NIH 3T3 fibroblasts and αT3 cells. Given the importance of STIM2 in facilitating SOCE, it may be an attractive target for SOCE inhibition in diseases associated with enhanced SOCE. For example, several cancers have been associated with upregulated expression of STIM1, STIM2, or Orai1 and increased SOCE [58,59]. STIM1 has a tumor growth promoting role in patients with breast cancer and cervical cancer [54,60]. In a genome-wide gene expression analysis of 20 primary glioblastoma samples, *Stim2* expression was upregulated [61]. Similarly, knockdown of STIM1 or Orai1 in rat and human glioblastoma cells inhibited tumor cell proliferation and promoted apoptosis [62]. Caution must be taken, however, when targeting STIM2 for therapeutic intervention in disease. Downregulation of *Stim2* expression is not always associated with decreased SOCE. In HT29 colon cancer cells, SOCE was significantly enhanced despite reduced *Stim2* expression [32]. This is likely a result of changes increased expression of TRPC1, Orai1, and STIM1. In comparison, reduction of *Stim2* in normal mucosal cells reduced SOCE and Ca^2+^ store content and promoted apoptosis resistance, suggesting a role for downregulated *Stim2* expression in tumor cell survival. Therefore, therapeutic interventions using *Stim2* as a molecular target are likely going to be cell type-specific and attention to effects of modifying *Stim2* expression on other proteins that regulate SOCE will be required. 

## 4. Materials and Methods 

### 4.1. Cell Culture

NIH 3T3 cells were obtained from ATCC and cultured at 37 °C with 5% CO_2_ in Dulbecco’s Modified Eagle’s Medium with 4.5 g/L d-glucose and 110 mg/L sodium pyruvate (DMEM (1X) + GlutaMAX-I, Thermo Fisher Scientific, Waltham, MA, USA) supplemented with 10% fetal bovine serum (FBS; Gibco), 100 U/mL penicillin and 100 μg/mL streptomycin . Cells exhibited fibroblast-like morphology and cell health was routinely assayed for mycoplasm contamination and normal rate of proliferation. Neuroendocrine αT3 cells were kindly provided by Dr. Richard J. H. Wojcikiewicz (SUNY Upstate Medical University, Syracuse, NY, USA) and were cultured with the same growth medium supplemented with 5% FBS. 

### 4.2. Cell Lysis, Immunoblotting, and Co-Immunoprecipitation 

Cells were removed from 10 cm plates with 155 mM NaCl, 10 mM HEPES, and 1 mM EDTA, pH 7.4, centrifuged at 1200 rpm for 2 min, and the cell pellet was disrupted for 30 min at 4 °C with ice cold lysis buffer (150 mM NaCl, 50 mM Tris-HCl, 1 mM EDTA, 1% Triton X-100, 10 μM pepstatin A, 1 mM Perfabloc SC, 0.2 μM soybean trypsin inhibitor, 1 mM dithiothreitol, pH 8.0). The lysates were clarified by centrifugation at 16,000× *g* for 10 min at 4 °C and the supernatant was used immediately or stored at −80 °C until use.

For immunoblotting, protein lysates were mixed with loading buffer (50 mM Tris-HCl (pH 6.8), 100 mM DTT, 2% SDS, 0.1% Bromophenol Blue, 10% glycerol) and heated for 30 min at 37 °C. The proteins were separated by SDS-PAGE (8% gel) for 40 min at 260 V. Proteins were electrophoretically transferred to nitrocellulose membrane for 60 min, at 0.7 mAmp. The membrane was blocked for 30 min in Tris-buffered saline with Tween-20 (TBST) buffer containing 5% nonfat dry milk (NFDM-TBST), then immunoblotted with primary antibodies overnight at 4 °C with gentle rocking. The blots were incubated for 1 h at room temperature in horseradish peroxidase (HRP)-conjugated secondary antibody. Immunoreactivity was detected with SuperSignal West Pico Chemiluminescent Substrate (Thermo Fisher Scientific, Waltham, MA, USA) and quantitated using a Bio-Rad Gel Documentation system with Quantity One software. 

Commercially available antibodies were used in our immunoblots: anti-STIM2 at 1:500 (Cell Signaling Technology, Danvers, MA, USA, Cat. No. 4917), anti-STIM1 at 1:500 (BD Biosciences, Franklin Lakes, NJ, USA, Cat. No. 610954), anti-Orai1 at 1:1000 (Proteintech Group, Inc., Rosemont, IL, USA, Cat. No. 13130-1-AP), anti-β actin at 1:1000 (Sigma-Aldrich Corp., St. Louis, MO, USA, Cat. No. A5316), anti-rabbit IgG(HRP) at 1:20,000 (Abcam, Cambridge, UK, Cat. No. ab6721), and anti-mouse IgG(HRP) at 1:10,000 (Abcam, Cat. No. ab6728). Antibodies were validated for specificity using knockdown and overexpression of the protein of interest. Additionally, the STIM2 antibody was used for coimmunoprecipitation and STIM2 was identified in the lysate by mass spectrophotometry. 

For co-immunoprecipitation experiments, cells were lysed with 1 ml ice-cold CHAPS lysis buffer (50 mM Tris-Base, 150 mM NaCl, 1 mM EDTA, 1% CHAPS (pH 8.0)) with 1 mM dithiothreitol, 25 μM pepstatin A, 0.2 μM soybean trypsin inhibitor, and 0.2 mM phenylmethylsulfonyl fluoride (PMSF) added fresh before use. For some studies, cells were exposed to 20 μM cyclopiazonic acid (CPA) in normal culture media for 6 min immediately prior to cell lysis. The lysates were collected and centrifuged at 13,000 rpm (Eppendorf 5415D microcentrifuge) for 10 min at 4 °C. The protein lysates were incubated for 7 h at 4 °C with 2.5 μg of STIM1 antibody (BD Bioscience catalog No. 610954). The antigen-antibody complex was immobilized on protein A-agarose beads (50 μL of 50% beads aqueous slurry, Thermo Scientific) for 16 h at 4 °C. The beads were washed four times with 500 μL of CHAPS lysis buffer and pelleted by centrifugation at 13,000 rpm for 30 s at 4 °C. The beads were resuspended with 30 μL of 2× SDS sample buffer (final concentrations: 25 mM Tris, 192 mM glycine, 1% SDS (pH 8.3)). The samples were then heated at 37 °C for 30 min, centrifuged for 60 s at 13,000 rpm, and the supernatant was used for Western blotting as described above. 

### 4.3. Generation and Analysis of STIM2 Knock-Out (KO) Cell Lines

The CRISPR-Cas9 system was used to target exons in the mouse *Stim2* gene. Oligonucleotides targeting exon 2 (5′-CAAGGACGGCGGGATCGAAG-3′) were annealed and ligated into AflII-linearized gRNA vector (Addgene, Cambridge, MA, USA). Alternatively, exon 8 (5′-GATGCAGCTAGCCATCGCTA-3′) was targeted to confirm our findings from our exon 2 KO. CRISPR target sequences were searched using NCBI BLAST to verify specificity. Following molecular cloning to insert our target sequences, the CRISPR gRNA vectors were sequenced for verification. Lipofectamine 2000 was used to transfect cells with a mixture of the *Stim2*-targeted gRNA construct, and vectors encoding hCas9 (Addgene, Cambridge, MA, USA) and EGFP-c1. In the αT3 cells, the same exon 2 target sequence was used, but cloned into a polycistronic vector (pCas-Guide-EF1a-GFP) that also contained hCas9 and GFP (OriGene, Rockville, MD, USA, Cat. No. GE100018). In the polycistronic vector we also cloned in a 20 base-pair scramble sequence to generate controls (5′-GTCGCTTGGGCGAGAGTAAG-3′). Two days post-transfection, EGFP-expressing cells were selected by fluorescence-activated cell sorting and plated one cell per well in a 96-well plate. Colonies were expanded and screened for knock-out of STIM2 expression by Western immunoblotting with anti-STIM2 (Cell Signaling Technology, Danvers, MA, USA, Cat. No. 4917) as described above. 

### 4.4. Cytoplasmic Ca^2+^ Imaging 

[Ca^2+^]_c_ was measured using Fura-2. Cells were grown on glass coverslips and incubated 15 min at room temperature with 1 μM Fura-2 acetoxymethylester (Invitrogen Corp.Carlsbad, CA, USA,), 0.01% (*v*/*v*) Pluronic^®^ F-127 in standard extracellular solution (SES) (NIH 3T3 cells) or Krebs-Ringer-HEPES buffer (KRH2) (αT3 cells). SES contained (in mM): 140 NaCl, 4 KCl, 1 CaCl_2_, 2 MgCl_2_, 1 KH_2_PO_4_, 10 HEPES-NaOH (pH 7.4), and 10 d-glucose. KRH2 contained (in mM): 140 NaCl, 4.7 KCl, 2.5 CaCl_2_, 1.2 MgSO_4_, 1.2 KH_2_PO_4_, 10 HEPES-NaOH (pH 7.4) and 2 d-glucose. Cells were placed into a temperature-controlled microperifusion chamber of an inverted fluorescence microscope (Olympus IX81, Olympus America, Inc., Center Valley, PA, USA). Cells were continuously superfused with SES or KRH2 (2 mL/min) at 37 °C. Fura-2 excitation wavelengths were 340 nm and 380 nm. The fluorescence emission intensities detected at 510 nm during excitation of Fura-2 at 340 nm (FI340) and 380 nm (FI380) were measured. MetaFluor software (Version 7.0, Molecular Devices LLC., San Jose, CA, USA) was used for image acquisition and analysis. Background fluorescence was subtracted from each FI340 nm and FI380 nm image. FI340/FI380 ratios were normalized to the average resting ratio 60 s prior to application of the stimulus. 

### 4.5. Flex Station

The SOCE inhibitor studies and NMG experiments were performed in a 96-well format in a FlexStation 3 multi-mode microplate reader (Molecular Devices, LLC., San Jose, CA, USA). NIH 3T3 cells (40,000 per well) and αT3 cells (50,000 per well) were seeded in a black-walled 96-well plate. Twenty-four hours later, cells were loaded with Fura-2 for 30 min at room temperature (SES, 1 μM Fura-2, 0.01% Pluronic^®^ F-127). The loading solution was removed and replaced with SES. [Ca^2+^]_c_ measurements were made using a FlexStation 3 plate reader controlled with SoftMax Pro software (Molecular Devices). Cells were excited at 335 nm and 375 nm, and the emitted light was detected at 505 nm using a 435-nm dichroic mirror. Test solutions were added to individual wells using the automated injection function of the FlexStation 3 at time points indicated in the figures. Data are expressed as the ratio of FI335/FI375 or relative FI335/FI375 (fold-change in FI335/FI375 relative to basal, resting FI335/FI375).

### 4.6. ER Ca^2+^ Imaging

Cells were transfected with a plasmid construct encoding D1ER, a fluorescence resonance energy transfer (FRET)-based indicator of [Ca^2+^]_ER_, by electroporation (Neon Transfection System, Thermo Fisher Scientific, Waltham, MA, USA). A suspension of NIH 3T3 cells (5 × 10^6^ cells/mL) was electroporated at 1350 V with two 20 ms pulses. Parameters for the αT3 cell electroporation were 1500 volts, 1 pulse, 20 ms. Cells were seeded and cultured on glass coverslips for 48 h at 37 °C, placed into a heated microperifusion chamber mounted on the stage of an inverted microscope (Olympus IX81) equipped with a charge-coupled device camera (ImagEM X2-CCD, Hamamatsu Photonics, Bridgewater, NJ, USA). Cells were superfused with SES or KRH2 at 37 °C and visualized with a 40× oil immersion objective. D1ER was excited at 440 nm and emission intensities were measured at 485 nm (FRET donor) and 535 nm (FRET acceptor). MetaFluor software (Molecular Devices LLC., San Jose, CA, USA) was used for image acquisition and analysis. Data are expressed as the fold-change in ratio of FRET acceptor and donor emission intensities (FRET FI535/FI485) normalized to resting, unstimulated values.

As an alternative strategy to indirectly measure ER Ca^2+^ store content, NIH 3T3 cells on coverslips were loaded with Fura-2 and images were acquired as described above. A SES/0Ca^2+^ buffer with 10 µM EGTA was perifused onto the coverslip before the addition of ionomycin to eliminate extracellular Ca^2+^ influx. Ionomycin was added to a final concentration of 2 µM and the subsequent change in [Ca^2+^]_c_ was measured. 

### 4.7. FRET Measurements of Protein-Protein Interactions

To measure the interaction of STIM1 and Orai1, cells were co-transfected with YFP-STIM1 (FRET acceptor) and CFP-Orai1 (FRET donor) using electroporation as described above. Quantitative FRET imaging was performed as described above. The WT cells served as our biological control, showing differences in the magnitude of FRET between Orai1-CFP and STIM1-YFP in WT cells compared to KO2-1. Additionally, a vehicle control (DMSO) was used to show no change in FRET when perifused onto cells instead of CPA. Data are expressed as the fold-change in the background subtracted ratio of FRET acceptor and donor emission intensities (FRET FI535/FI485 nm) normalized to resting values. Resting FI535/FI485 values in KO2-1 and WT were not different.

### 4.8. Morphometric Analysis of STIM1 Localization

Cells were transfected with YFP-STIM1 in WT and KO2-1 cells. To reconstitute STIM2 expression in KO2-1 cells, YFP-STIM1 was co-expressed with CFP-STIM2. Two days post-transfection, a time-lapse series of images were acquired in which cells were perifused with test solutions at 37 °C. Cells were initially bathed in normal, 1 mM Ca^2+^ extracellular solution to establish a baseline distribution, and then superfused with a Ca^2+^-free solution supplemented with 20 μM cyclopiazonic acid (CPA) to discharge and prevent refilling of intracellular Ca^2+^ stores. To restore ER Ca^2+^ levels, cells were bathed in the normal extracellular solution without CPA. Confocal images acquired immediately before the reconstitution of extracellular Ca^2+^ and CPA washout were background subtracted and processed using 2D deconvolution. Morphometric analysis was used to count the number and determine the area of individual puncta in each cell (MetaMorph^®^ Microscopy Automation & Image Analysis Software, Version 7.0, Molecular Devices, LLC., San Jose, CA, USA).

### 4.9. Reagents

Fura-2 acetoxymethylester and ionomycin were purchased from Thermo Fisher Scientific and CalBiochem, respectively. ML-9 and SKF96365 hydrochloride were obtained from Sigma-Aldrich Co. (St. Louis, MO, USA). [2,6-difluoro-*N*-(1-[4-hydroxy-2-(trifluoromethyl)benzyl]-1*H*-pyrazol-3-yl)benzamide] (GSK-7975A) was obtained from Glaxo Laboratories Ltd. (Greenford, UK). *N*-arachidonoyl glycine (NAGly) was purchased from Cayman Chemical (Ann Arbor, MI, USA). Cyclopiazonic acid (CPA) was obtained from Millapore Sigma. Orai1-E106A, CFP-Orai1 and YFP-STIM2 were obtained from Addgene.org. YFP-STIM1 was a generous gift from Tobias Meyer (Stanford University). D1ER was provided by the late Roger Y. Tsien (University of California San Diego). 

### 4.10. Statistical Analysis

Our data was analyzed and revealed nearly normal to normal distribution, equal variance, and were independent measurements; therefore, we used unpaired Student’s *t*-test assuming equal variance for intergroup comparisons (*p* < 0.05 was considered statistically significant), as detailed in the figure legends. Data traces are plotted as the mean ± SEM. Box and whisker plots show mean (open square), median (solid line), 25th and 75th percentiles (box), and minimum and maximum measurements (whiskers).

## Abbreviations

STIM	Stromal Interaction Molecule
Ca^2+^	Calcium
ER	Endoplasmic Reticulum
PM	Plasma Membrane
